# Lifestyle, psychological and demographic predictors of anxiety: insights from a large-scale survey and machine learning analysis

**DOI:** 10.3389/fpsyt.2026.1727014

**Published:** 2026-05-20

**Authors:** Dur E Nishwa, Zeeshan Abbas, Seung Won Lee

**Affiliations:** 1 Department of Precision Medicine, School of Medicine, Sungkyunkwan University, Suwon, Republic of Korea; 2 Department of Biomedical Engineering, College of IT Convergence, Gachon University, Seongnam, Republic of Korea; 3 Department of Artificial Intelligence, Sungkyunkwan University, Suwon, Republic of Korea; 4 Department of Metabiohealth, Sungkyunkwan University, Suwon, Republic of Korea; 5 Personalized Cancer Immunotherapy Research Center, School of Medicine, Sungkyunkwan University, Suwon, Republic of Korea; 6 Department of Family Medicine, School of Medicine, Kangbuk Samsung Hospital, Sungkyunkwan University, Seoul, Republic of Korea

**Keywords:** anxiety, lifestyle factors, machine learning, sleep duration, stress

## Abstract

**Objective:**

Anxiety is influenced by a combination of lifestyle, psychological, and demographic factors. This study aimed to evaluate these associations and explore the potential of machine learning in predicting anxiety severity.

**Methods:**

Anxiety levels were evaluated using a large survey-based dataset of 11, 000 adults alongside demographic, physiological, and psychological measures. Descriptive statistics and inferential analyses were conducted in IBM SPSS to identify associations between key variables. Several machine learning regression algorithms, including linear, regularized, and ensemble models, were implemented in Python to predict anxiety levels. Model performance was evaluated using standard error metrics.

**Results:**

Our findings revealed significant associations of anxiety with stress and sleep duration, while demographic attributes such as family history of anxiety and occupation also influenced outcomes. Ensemble machine learning algorithms achieved superior performance compared to single and linear-model approaches. Feature importance analysis identified stress, sleep, and caffeine intake as top predictors of anxiety.

**Conclusions:**

The integration of statistical approaches with machine learning applications highlights the multifactorial nature of anxiety and demonstrates the potential of predictive modeling in mental health care. Future research should emphasize longitudinal designs and the incorporation of biological and digital markers to enhance clinical applicability and prediction.

## 1 Introduction

Anxiety disorders are among the most prevalent mental health conditions globally and are frequently associated with heightened individual stress, situation-specific perceived threats, impaired social functioning, and an elevated risk of both physical and psychiatric comorbidities (1, 2). They often emerge during adolescence and early adulthood, typically between the ages of 10 and 24, and are linked to significant adverse effects on health and functional role performance (3–5). Global data reported a growing incidence of anxiety disorders among young people, with studies indicating a 52% increase in global prevalence between 1990 and 2021, particularly among those aged 10–24 years. Studies also reported a sharp rise in anxiety during the COVID-19 era from 2019 to 2021 (4). Clinically, anxiety disorders manifest through a variety of psychological and physical symptoms. Psychological symptoms commonly include restlessness, excessive worry, difficulty concentrating, and irritability, whereas physical symptoms often involve sleep disturbances, muscle tension, rapid heart rate, and gastrointestinal distress (6, 7). In some cases, individuals experience symptoms characteristic of panic disorder, which, when perceived as threatening can trigger psychophysiological responses such as sweating, dizziness, and increased heart rate (8). If left untreated, these symptoms may progress to chronic anxiety, increasing the risk of comorbid conditions including hypertension, dementia, and cardiovascular disease (9, 10). Existing literature identifies the combination of psychotherapy and medication as a typical treatment option for anxiety. The psychological issues that cause anxiety require psychotherapy, whereas physiological problems need to be addressed with pharmacological methods (11, 12).

### 1.1 Lifestyle, physiological, and psychological influences on anxiety

Several studies have identified a wide range of factors that contribute to the onset and maintenance of anxiety. These include lifestyle behaviors such as sleep patterns, alcohol consumption, smoking, caffeine intake, and certain physical activities, which are frequently associated with the emergence of anxiety symptoms. Sleep disturbances, particularly insufficient sleep or insomnia, are consistently linked with elevated symptoms of anxiety (13, 14). For instance, a large-scale cross-sectional study conducted among Chinese commercial pilots revealed that both insufficient sleep (less than 7 hours) and poor sleep quality were significantly associated with an increased risk of anxiety (15). In a repeated-measures study among Chinese university students, an increased incidence of anxiety was observed among those experiencing poor sleep quality over time, with cognitive emotion regulation emerging as a key mediator (β = 0.40, p <.001) (16). In parallel, a recent 2024 meta-analysis confirmed that caffeine intake, although widely consumed, is linked to provoking anxiety symptoms, and higher doses are more strongly associated with anxiety-related outcomes, even in non-clinical samples (17, 18). Another large-scale survey conducted in Korea in 2025 demonstrated that excessive consumption of drinks containing high levels of caffeine was associated with a higher prevalence of anxiety in both male and female adults (19). Furthermore, smoking, which is common among individuals with elevated anxiety, is thought to serve as a physiological contributor to sustained anxiety through nicotine dependence (20, 21). Physical activity has also been identified as a protective factor against anxiety. A 2021 cohort study found that regular exercise is linked to a lower incidence of anxiety disorders (22), Similarly, a study conducted in 2025 observed that increased frequency of physical activity is associated with improved sleep quality and a reduced incidence of anxiety traits, particularly among young individuals (23).

On the physiological side, several factors play a central role in anxiety. Existing literature indicates that individuals with chronic anxiety disorders show signs of autonomic nervous system hyperactivity, characterized by an increased breathing rate and elevated heart rate (6). A 2024 meta-analysis confirmed the association between heart rate variability (HRV), a marker of autonomic function, and anxiety disorders, reporting reduced HRV in individuals with anxiety, thereby supporting its role as a physiological biomarker (24). Another study conducted in 2024 validated HRV’s sensitivity for detecting anxiety using wearable devices in real-world situations (25).

Finally, psychological variables particularly perceived stress are considered among the most robust predictors of anxiety (26). Frequent exposure to stressful life events, especially those involving loss, humiliation or embarrassment, entrapment, and danger, has been shown to significantly increase the risk of anxiety onset (27). Systematic reviews have demonstrated that stress reduction is associated with increased HRV and a reduction in anxiety symptoms across diverse populations (28). Collectively, these findings emphasize the complex interaction between lifestyle, physiological, and psychological domains in shaping susceptibility to anxiety.

### 1.2 Gaps in the literature

While each of these factors lifestyle, physiological, and psychological has been extensively linked to anxiety, most research has examined them within a single domain or in small, homogenous clinical samples, limiting comprehensive understanding. For example, a large nationwide survey conducted in the Chinese adolescent population focused on lifestyle behaviors and mental health, observing that lower anxiety ratios were dominant in adults who followed healthy lifestyle habits, however, perceived stress and physiological markers were not included in the study (29). Similarly, Dabravolskaj et al. (30) conducted a prospective analysis on Canadian young adults and found that healthy lifestyle adherence was associated with lower anxiety scores, but the sample was limited to young adults, and physiological or stress-related predictors were not assessed (30). Although experimental interventions such as improving sleep hygiene and lifestyle modification clearly reduced anxiety symptoms in a randomized trial summarized by Amiri et al. (2024), they did not evaluate psychological stress and physiological arousal in the same individuals (31). In sum, existing research typically focuses on examining one or two domains at a time, often in specific populations such as convenience samples or adolescents, and seldom compares lifestyle behaviors alongside physiological and psychological predictors within the same large sample. These approaches limit our ability to evaluate which domains carry more predictive power for anxiety severity and reduce the generalizability of findings to diverse adult populations.

In parallel, recent ML and AI approaches to anxiety have mainly focused on predicting clinically significant anxiety or classifying symptom severity using questionnaire−based measures. During the COVID−19 pandemic, several studies applied supervised machine learning to self−report scales such as the DASS−21 to predict stress, depression, and anxiety in community samples, and used electronic health records and survey−based predictors to model diagnoses of generalized anxiety disorder and related condition (32, 33). Other studies have applied in−lab data and multimodal physiological signals, or reviewed in−the−wild sensing approaches and broader ML pipelines for automated anxiety detection (34–36). In contrast, fewer studies have jointly examined lifestyle, psychological, psychosocial and demographic predictors of anxiety within the same large community dataset while systematically benchmark multiple regression−based ML models for predicting continuous anxiety severity.

### 1.3 Objectives and hypotheses

This study aims to analyze the influence of lifestyle, psychological, and demographic factors on anxiety within a large adult sample. Specifically, it examines whether psychological variables such as perceived stress and major life events show stronger associations with anxiety compared to lifestyle. Our dataset did not distinguish between different types of anxiety, therefore, all predictors were examined against a single outcome, the continuous self−reported anxiety severity score. Guided by previous epidemiologic and ML studies that highlight the roles of sleep, stress, health behaviors, and physiological and psychosocial factors in anxiety (37, 38) we selected predictors spanning four domains: lifestyle, physiological/autonomic markers, psychosocial and clinical variables and demographics. Furthermore, we anticipate that ensemble machine learning models will yield more accurate predictions of anxiety compared to linear and non-linear single-model approaches.

## 2 Method

### 2.1 Participants and sampling

This study analyzed an open-access dataset, originally compiled by Zhang Chenyuan (2024) and hosted on the Kaggle platform (https://www.kaggle.com/datasets/natezhang123/social-anxiety-dataset/data). The dataset contains 11, 000 participants comprising diverse age groups, demographic backgrounds, and occupations, exhibiting the broad representation of the general adult population. The dataset comprises survey-based and observational data reflecting lifestyle behaviors, demographic characteristics, physiological measures, psychological factors, and clinical/psychosocial variables. The dataset is not associted to a specific country, region or sampling frame, and therefore should be interpreted as representing a heterogeneous population rather than a nationally representative sample. Inclusion criteria included adults 18 years or older with complete information for all relevant measures containing anxiety level, perceived stress, lifestyle habits, physiological measures, and additional psychosocial indicators. All 11, 000 individuals were retained for final analysis, as the dataset contained no missing values.

### 2.2 Measures and variables

The primary outcome of interest was anxiety, assessed using a single 10−point self−report item ranging from 1 (no anxiety) to 10 (extreme anxiety). This measure reflects participant’s perceived anxiety severity and does not specify any particular anxiety disorder, it should therefore be interpreted as a dimensional index of self−reported anxiety rather than a diagnostic measure. For categorical analyses, anxiety scores were grouped into low (1–3), moderate (4–6), and high (7–10) anxiety.

Lifestyle behaviors included smoking, alcohol consumption, sleep duration, caffeine intake, physical activity and diet quality. Smoking status was obtained from the ‘Smoking’ variable, coded as a binary response (Yes/No), participants coded as “Yes” classified as smokers and those coded “No” as non-smokers. Alcohol intake was derived from the ‘alcohol consumption (drinks/week)’ variable and grouped into none (0 drinks/week), occasional (1–7 drinks/week), and frequent (>7 drinks/week). Sleep duration was categorized <6 hours as low, 6–8 hours as moderate, and >8 hours as high. Caffeine intake categorized into low (0–100 mg/day), moderate (101–300 mg/day) and high (>300 mg/day). Physical activity was derived from the ‘Physical Activity (hrs/week) variable, which reflects self-reported total weekly hours of any physical activity. Because the dataset does not differentiate specific activity types (e.g., walking, exercise or sports), it was analyzed as overall physical activity time and categorized as low (0–2 hrs/week), moderate (2–5 hrs/week), and high (5–9 hrs/week). Diet Quality was evaluated using the ‘Diet Quality(1-10)’ index, where higher scores indicate a heathier overall diet. This index was categorized into poor (1–3 scores), moderate (4-7), and healthy (8-10). As the dataset does not provide item−level dietary intake, this variable represents a synthetic diet quality indicator rather than a validated dietary instrument.

Physiological measures included self-reported breathing rate (breaths per minute) and resting heart rate (beats per minute, bpm), both treated as continuous variables, alongside sweating and dizziness severity levels, each measured on a 10-point scale to capture autonomic symptom burden. The primary psychological factor was perceived stress, assessed using a 10-point scale (1= no stress, 10=extreme stress). Psychosocial and clinical variables comprised current medication use, family history of anxiety, participation in therapy sessions, and recent major life events. Medication use was derived from the binary ‘Medication’ variable (Yes/No), indicating the current use of any medication at the time of survey, information regarding medication type, dosage or duration was not available. Family history of anxiety assessed using the binary response (Yes/No), with a positive history defined as a “Yes” response indicating at least one affected family member. Participation in therapy was assessed via ‘Therapy Sessions per month’, indicating the number of mental−health–related therapy sessions attended per month, for descritive purposes, we categorized participation as none (0 sessions/month), occasional (1–3 sessions/month), and regular (≥4 sessions/month), though the dataset does not specify therapy modality or setting. Recent major life events were captured using the ‘Recent Major Life Event’ variable (Yes/No), a “Yes” response indicates at least one recent major life event, although the dataset does not specify event type or exact time frame.

Demographic features included age (years), gender (male, female, other), and occupation. Occupation status was measured using the categorical ‘Occupation’ variable, indication self reported roles such as artist, student, nurse, teacher, doctor, scientist, engineer, and other professional categories and was analyzed according to these categories as provided in the dataset.

### 2.3 Procedures

All analysis was conducted using IBM SPSS version 27 and Python 3.10. Predictors were categorized into three domains comprising lifestyle, physiological, and psychological, while psychosocial and clinical parameters were analyzed in conjunction with these domains.

### 2.4 Statistical analysis

Descriptive statistics, including means, standard deviations, percentages, and frequencies, were calculated for all variables. To compare mean anxiety scores among binary predictors such as gender, smoking status, recent life events, and family history of anxiety, independent-samples t-tests were conducted, while one-way analyses of variance (ANOVA) were employed for predictors with three or more categories (e.g., occupation, caffeine intake, therapy participation, diet quality, and physical activity). Spearman’s rank correlation coefficients were calculated for non-normally distributed continuous variables such as stress, heart rate, breathing rate, sweating, and dizziness with continuous anxiety scores. To examine associations between categorical predictors and anxiety categories, Chi-square test was employed.

### 2.5 Machine learning analysis

To complement the statistical approaches, machine learning regression algorithms were evaluated for their predictive performance in identifying the most suitable model for predicting anxiety using the full set of predictors. The analysis was conducted in Python 3.10 with the scikit-learn library. Predictors encompassed demographic, physiological, psychosocial, lifestyle, and clinical domains. Seven regression models were trained and compared including Gradient Boosting Regressor, Random Forest Regressor, Lasso Regression, Linear Regression, Elastic Net Regression, Support Vector Regression (SVR), and Multilayer Perceptron Regressor (MLP). These models were selected to represent a range of linear, regularized, ensemble-based, and neural network approaches. Model performance was assessed using five common metrics: Mean Absolute Error (MAE), Mean Absolute Percentage Error (MAPE), Root Mean Square Error (RMSE), coefficient of determination (R²), and cross-validated R² (CV R²). Lower MAE, MAPE, and RMSE values indicate better predictive accuracy, whereas higher R² and CV R² values reflect stronger generalization and explanatory power. The dataset was randomly split into a training set (80%) and a held-out test set (20%) using a fixed random seed. Within the training set, 5-fold cross-validation was applied for hyperparameter tuning and to estimate cross-validated R² for each algorithm. Final performance metrics reported are based on predictions generated for the independent test set. To facilitate comparison across models, metrics were normalized to a 0–1 scale, with values closer to 1 indicating superior performance.

## 3 Results

### 3.1 Participant demographics and distribution of key variables

A total of 11, 000 participants were included in this analysis. The mean age of the sample was 40.2 years (SD = 13.2; range = 18–64). The age distribution of participants was approximately uniform between 20 and 60 years, representing an adult population.

#### 3.1.1 Psychological variables

Regarding mental-health related measures, participants reported mean anxiety and stress scores of 5.14 (SD = 2.87) and 5.86 (SD = 2.93) respectively, on a 10-point scale. Anxiety showed a mild peak around scores of 3–4, tapering off at higher severity levels, whereas stress levels were more broadly distributed, with higher frequencies in the upper range (scores 7–10).

#### 3.1.2 Physiological measures

Mean heart rate was 90.9 bpm (SD = 17.3), with a relatively uniform distribution spanning 60–120 bpm. Breathing rate averaged 21 breaths per minute (SD = 5.2), also displaying a uniform spread across the physiological range (12–29 breaths/min). Caffeine intake averaged 286 mg/day (SD = 145), with a broad, multimodal distribution, while alcohol consumption averaged 9.7 drinks per week (SD = 5.7) indicating diverse consumption habits across participants.

#### 3.1.3 Lifestyle behaviors

Dietary quality (mean = 5.18, SD = 2.90, scale 1–10) was fairly evenly distributed, with slight clustering around lower scores, suggesting suboptimal dietary habits in a considerable proportion of the sample. Participants reported an average of 6.65 hours of sleep per night (SD = 1.23) and 2.94 hours of physical activity per week (SD = 1.83). Sleep duration followed a near-normal distribution, with most individuals reporting between 6 and 8 hours per night. Very few people sleep less than 4 or more than 9 hours, indicating that extreme sleep patterns are rare as majority of participants maintained typical sleep patterns. Physical activity showed a positively skewed distribution, with the majority of individuals engaging in 1–4 hours of activity per week. Few participants reported more than 6 hours of weekly activity, indicating that high levels of physical activity were relatively rare.

**Figure 1:**
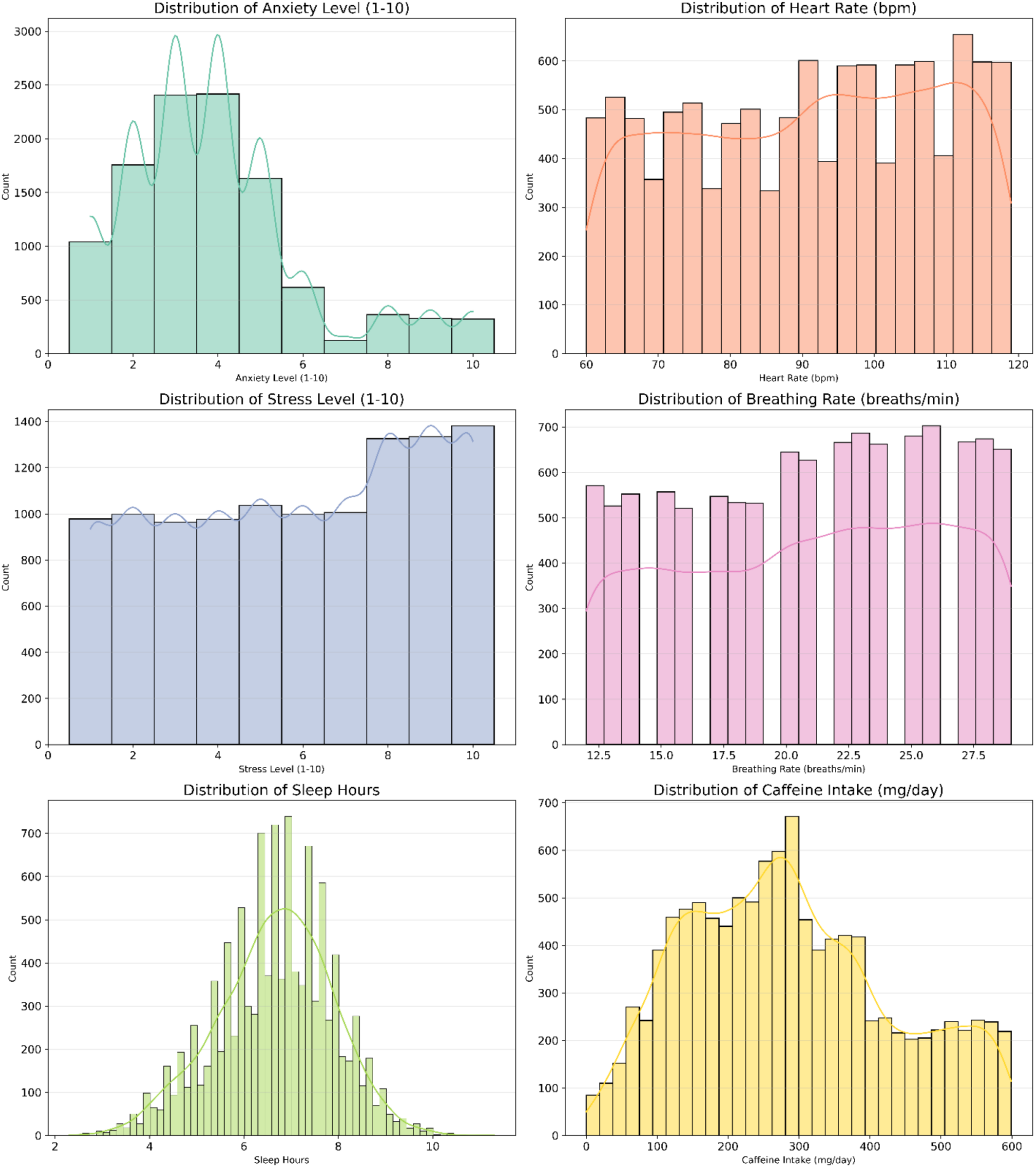
Distribution patterns of lifestyle, psychological, and physiological variables histograms with density curves display the frequency and distribution of anxiety, stress, sleep, heart rate, breathing rate, and caffeine intake among study participants.

Collectively, these distributions reflect the heterogeneity of lifestyle, psychological, and physiological characteristics in the study sample. Variables such as sleep duration, heart rate and breathing rate exhibited relatively symmetric distributions, whereas others (e.g., caffeine intake, stress levels, anxiety levels, therapy sessions, and physical activity) showed marked skewness (**Figure 1**).

### 3.2 Anxiety levels by demographic and lifestyle factors

Our study sample did not observe any significant difference in anxiety scores between males and females. However, smokers, individuals with a family history of anxiety, and those who experienced a recent life event reported significantly higher anxiety levels. The strongest effect was observed for family history of anxiety (d = -0.39) (**Table 1**).

**Table 1:** Mean anxiety scores by demographic and lifestyle factors.

Variable	Group	Mean ± SD	t(df)	p-value	Cohen’s d	Interpretation
Gender	Female	3.93 ± 2.13	-0.04 (7385)	0.965	-0.001	No significant difference
	Male	3.93 ± 2.15				
Smoking Status	Smoker	4.09 ± 2.27	-8.68 (10, 958)	<.001	-0.164	Higher anxiety in smokers
	Non-smoker	3.75 ± 1.93				
Family History of Anxiety	Yes	4.31 ± 2.29	-20.69 (10, 874)	<.001	-0.390	Higher anxiety in those with family history
	No	3.50 ± 1.81				
Life Events	Recent event	4.09 ± 2.28	-8.26 (10, 838)	<.001	-0.157	Higher anxiety after recent life events
	No event	3.76 ± 1.93				

SD, standard deviation; t(df), t statistic with degrees of freedom; p−value, probability value; Cohen’s d, standardized mean difference effect size.

### 3.3 Anxiety levels by occupational and health-related factors

Anxiety levels varied significantly across occupational and health-related factors (**Table 2**). Lifestyle factors demonstrated stronger associations: individuals with high caffeine intake, poor diet quality, low physical activity, and those attending regular therapy sessions exhibited the highest anxiety scores. *Post hoc* comparisons confirmed significant pairwise differences across these groups (all p <.001). Moderate caffeine consumers also reported slightly higher anxiety levels compared to low consumers, the occasional therapy group had slightly higher anxiety than the no therapy group, participants with a moderate diet also reported significantly higher anxiety than those with a healthy diet, moderate activity participants reported higher anxiety than those with high activity. Occupation was associated with more modest differences, with lawyers and doctors reporting the highest anxiety compared to teachers and musicians among others, suggesting that occupational role is associated with varying levels of anxiety.

**Table 2:** Mean anxiety scores across occupational and health-related factors.

Factor	Groups (Mean ± SD)	F(df)	p-value
Caffeine intake	Low = 3.21 ± 1.9; Moderate = 3.40 ± 2.0; High = 5.54 ± 2.3	722.13 (2, 10, 997)	<.001
Therapy sessions	None = 3.21 ± 1.9; Occasional = 3.40 ± 2.0; Regular = 5.54 ± 2.3	1433.01 (2, 10, 990)	<.001
Diet quality	Healthy = 3.43 ± 1.9; Moderate = 3.87 ± 2.0; Poor = 4.50 ± 2.2	260.58 (2, 10, 997)	<.001
Physical activity	High = 3.37 ± 1.8; Moderate = 3.57 ± 2.0; Low = 4.64 ± 2.2	376.54 (2, 10, 984)	<.001
Occupation	Range: 3.75 (Teachers) – 4.21 (Lawyers)	5.08 (12, 10, 987)	<.001

SD, standard deviation; F(df), F statistic with degrees of freedom; p−value, probability value.

### 3.4 Correlation between anxiety and key predictors

**Figure 2:**
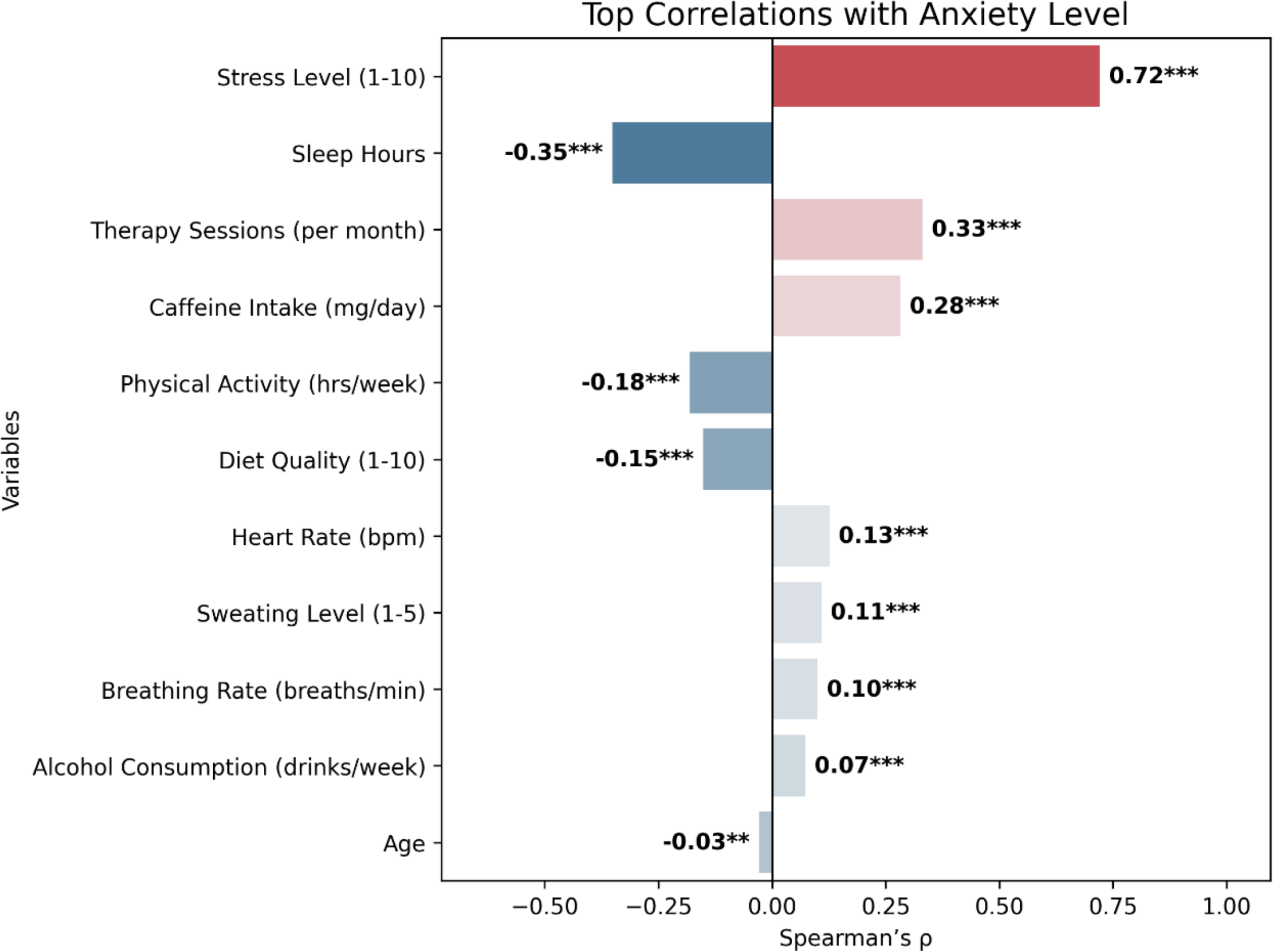
Ranked Spearman’s correlations with anxiety. Bar plot displays the strength and direction of correlations between anxiety and variables. Variables are ordered from strongest to weakest correlation. Positive correlations are shown in red, and negative correlations in blue. Stress level emerged as the strongest correlate, followed by sleep duration and caffeine intake ***p < 0.001.

Spearman’s rank-order correlation was conducted to evaluate associations between anxiety and various demographic, physiological, and behavioral variables. **Figure 2** presents the ranked Spearman’s correlation coefficients, illustrating the strength and direction of these associations. Overall, all examined associations were statistically significant (p <.001). A very strong positive correlation was observed between anxiety and stress level (rs = 0.721), indicating that stress is the strongest predictor in this dataset. Sleep duration (negative, rs = −0.351) and caffeine intake (positive, rs = 0.282) demonstrated moderate associations. Weaker correlations were observed with physical activity (negative, rs = −0.181), heart rate (positive, rs = 0.13), and breathing rate (positive, rs = 0.101). Alcohol consumption showed only a minimal positive relationship with anxiety (rs = 0.073), while age had a negligible negative correlation (rs = −0.029). When comparing lifestyle, physiological, and psychological domains, stress emerged as the most robust correlate of anxiety, followed by sleep duration, therapy sessions, and caffeine intake. Lifestyle behaviors, including alcohol consumption and physical activity, showed modest correlations; in contrast, physiological features such as heart rate and breathing rate exhibited consistent but weaker associations.

### 3.5 Correlation between anxiety and categorical factors

**Figure 3:**
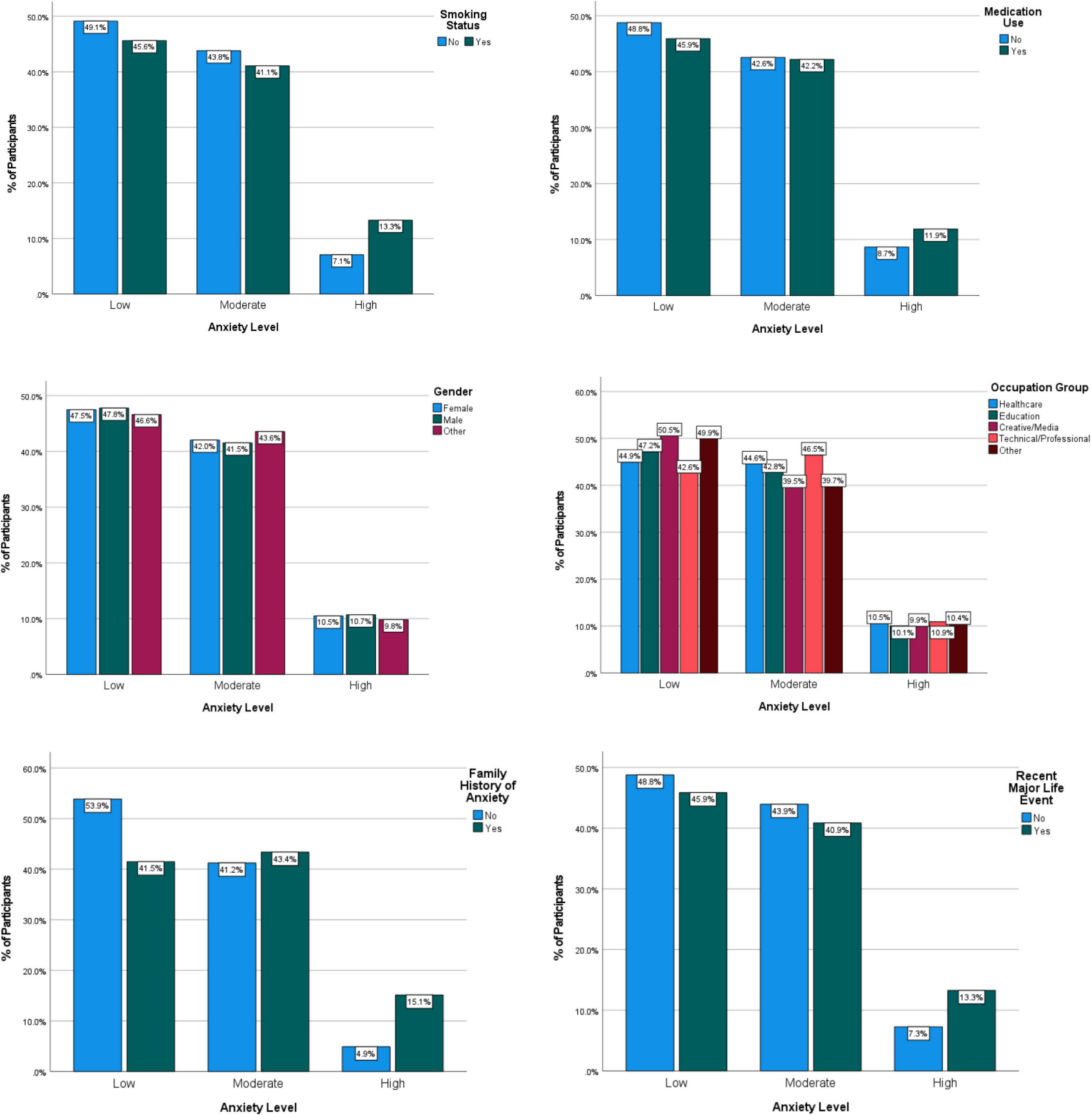
Association between categorical variables and anxiety levels. Stacked bar charts represent the distribution of participants across low, moderate, and high anxiety levels according to categorical predictors. The X-axis represents anxiety levels, while the Y-axis indicates the percentage of participants within each category. Factors examined include smoking status, medication use, gender, occupation group, family history of anxiety, and recent major life events.

Chi-square test was conducted to examine the association of categorical variables with anxiety. Several categorical variables exhibited significant associations with anxiety level. A significant association was observed between smoking status and anxiety, χ² (2, N = 11, 000) = 114.63, p <.001. High-anxiety participants were disproportionately smokers (67.5%), compared to 50.9% of those with moderate anxiety and 50.7% of those with low anxiety. Similarly, medication use was significantly associated with anxiety, χ²(2, N = 11, 000) = 32.31, p <.001, with a higher proportion of medication use reported among those with severe anxiety (59.3%), relative to moderate (51.3%) and low (50.0%) anxiety. Examining the sociodemographic characteristics, it was observed that gender was not significantly associated with anxiety, χ² (4, N = 11, 000) = 3.911, p = .418; anxiety levels were relatively evenly distributed across males, females, and other gender identities. In contrast, occupation was significantly associated with anxiety level, χ²(8, N = 11, 000) = 46.65, p <.001, as anxiety levels varied significantly by occupation, with participants in creative/media roles exhibiting the highest prevalence of anxiety (30.3%), followed by Technical/Professional roles (23.8%), while Education professionals showed the lowest prevalence (14.5%). Family history of anxiety emerged as one of the strongest correlates, χ²(2, N = 11, 000) = 369.89, p <.001, with participants with high anxiety more frequently reporting family history (77.8%) compared to low-anxiety participants (46.6%). Anxiety was also significantly associated with recent major life events, χ² (2, N = 11, 000) = 106.67, p <.001. Major life events were more frequently experienced by high-anxiety participants (65.6%) than those with low (49.6%) or moderate anxiety (49.3%) (**Figure 3**).

### 3.6 Predictive modeling outcomes

**Figure 4:**
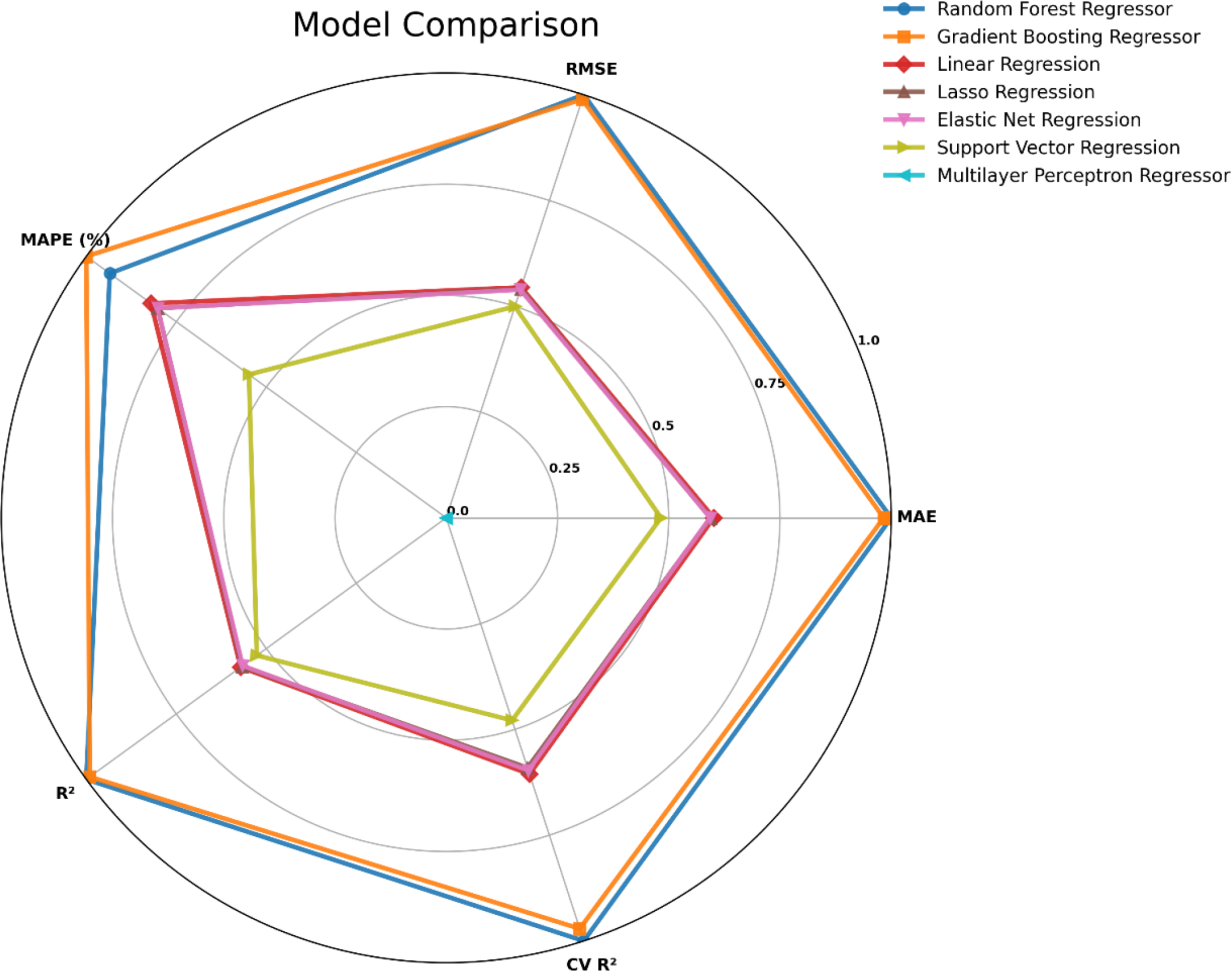
Comparative performance of machine learning models in predicting anxiety levels. The radar chart illustrates a comparative overview of various regression algorithms (Random Forest Regressor, Gradient Boosting Regressor, Linear Regression, Lasso Regression, Elastic Net Regression, Support Vector Regression, and Multilayer Perceptron Regressor) across five metrics, MAE, RMSE, MAPE, R², and CV R². Higher values indicate better performance after normalization. The visualization highlights the superior performance of Gradient Boosting and Random Forest across all evaluation metrics compared to linear and neural network models.

Machine learning models were trained to predict anxiety levels across various predictors. As observed in **Figure 4**, among multiple tested models, ensemble models (Gradient Boosting and Random Forest) outperformed linear (Linear, Lasso, Elastic Net) and nonlinear (Support Vector Regression, Multilayer Perceptron) models across all evaluation metrics, achieving the highest overall performance with consistently low error metrics (MAE, RMSE, and MAPE) and high R² values. Gradient Boosting, in particular, presented the most reliable and accurate performance in predicting anxiety levels in this dataset. Linear models exhibited relatively low predictive ability, while SVR showed moderate results. The least performance was observed with the MLP regressor, which had the lowest R² and highest error values.

**Figure 5:**
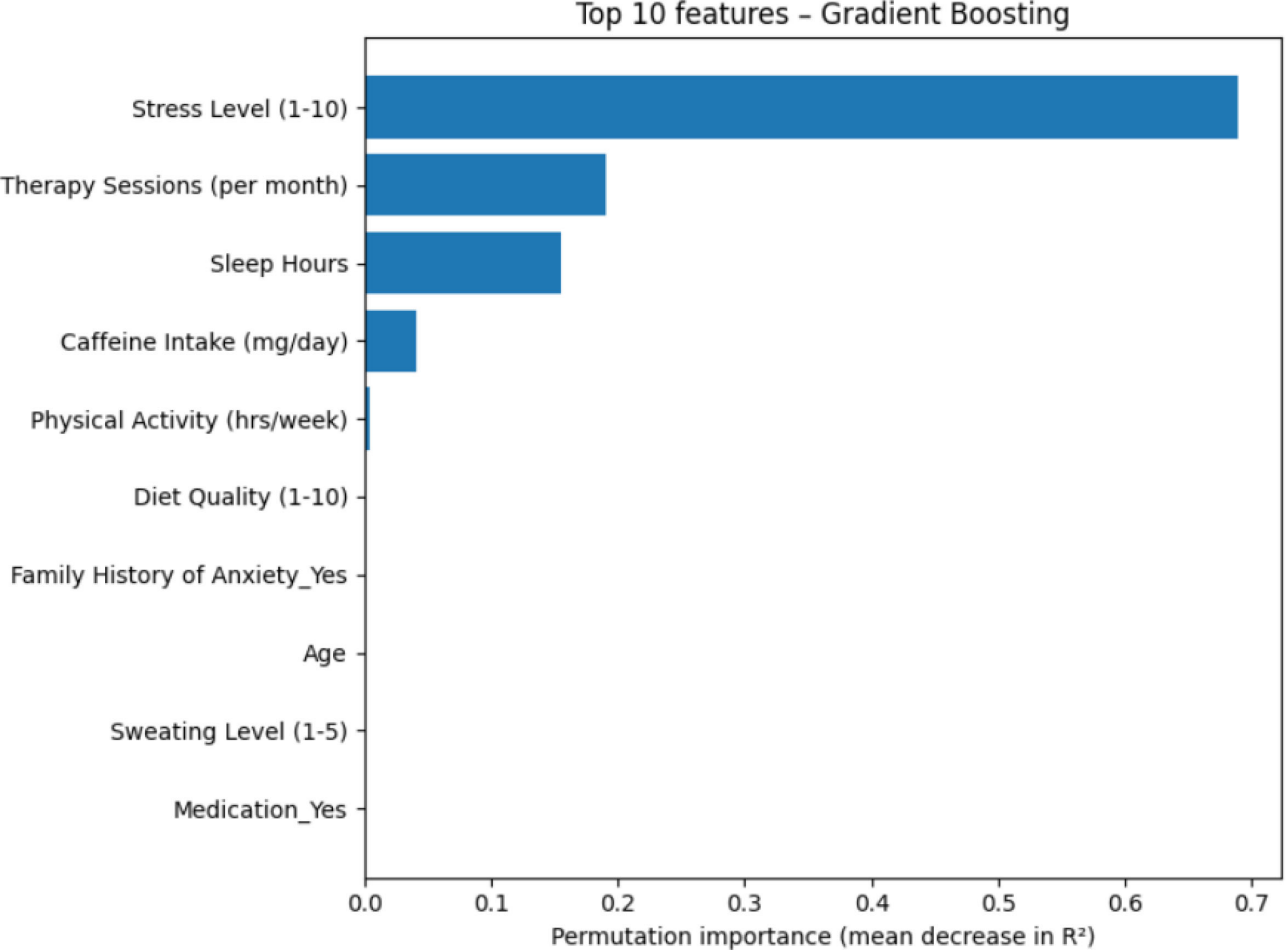
Top 10 predictors of anxiety in the gradient boosting model Bars show permutation importance, quantified as the mean decrease in R^2^ when each predictor is randomly permuted while all others are kept fixed. Higher values indicate a greater contribution of that predictor to overall model performance.

To aid interpretation, feature importance was evaluated for the best-performing Gradient Boosting Regressor using permutation importance to quantify each predictor’s contribution. As illustrated in **Figure 5**, a small set of predictors demonstrated markedly higher importance than others, with stress level, therapy sessions and sleep hours emerging as strongest candidates to model performance. In contrast, variables such as current use of medication and sweating level demonstrated relatively low importance, indicating a more limited role in predicting anxiety levels within this dataset.

## 4 Discussion

This study demonstrates predictors of anxiety in a large adult population by integrating statistical approaches with machine learning to examine the factors that contribute to anxiety outcomes. The findings revealed that several factors, including stress, smoking, family history of anxiety, major life events, sleep duration, and caffeine intake, are strongly associated with anxiety, while physiological measures such as breathing rate and heart rate exhibited consistent but weaker associations. Notably, the ensemble machine learning models, Gradient Boosting and Random Forest, outperformed other linear and nonlinear models, highlighting the value of predictive analytics in mental health research.

Previous machine learning studies on anxiety have primarily focused on classification of anxiety status, often achieving good to excellent performance. Recent studies by Priya et al. (32) and Albagmi et al. (33) used algorithms such as Random Forests and SVMs to predict anxiety, stress, and depression from questionnaire data, reporting accuracies above 90%. Comprehensive reviews by Tayarani−N and Shahid (38) and He et al. (36) summarize recent approaches for anxiety prediction and note that performance tends to be highest when rich digital features and intensive monitoring are available. In contrast, our study uses a compact set of self−reported demographic, lifestyle, psychosocial, and clinical variables to predict continuous anxiety scores, achieving moderate R^2^ values and comparatively low absolute errors on the test set. Although our performance is lower than that typically reported for physiology− or sensor−based systems, these findings suggest that useful prediction is still possible without high−cost sensing or longitudinal data. In contrast to studies using biological or digital phenotypes, which often exhibit high discrimination performance for anxiety detection, our self−report–based models showed only moderate R^2^ but relatively low absolute prediction errors, indicating useful yet more modest predictive performance.

Examining the demographic and lifestyle attributes of our study, we did not observe significant gender differences in our data, which contrasts with previous meta-analyses suggesting that women experience a significantly higher prevalence of anxiety compared to men (39, 40). A possible explanation is that our study sample presents a dataset with balanced gender representation and is more focused on the adult population (18–64 years), thereby minimizing the likelihood of such disparities. In contrast, smoking showed a strong association with anxiety. This aligns with a study that demonstrated a bidirectional relationship between cigarette smoking and anxiety symptoms, indicating that smoking may provide short-term relief yet exacerbates anxiety in the long term (41). Similarly, family history of anxiety presented as one of the strongest correlates of anxiety, proved by the previous studies demonstrated that anxiety disorders have significant relation with familial aggregation (42). The occurrence of recent major life events reinforces stress-related psychological symptoms and also acts as a predictor of anxiety outcomes, while acute psychosocial stress serves as a trigger for anxiety development among vulnerable individuals (43, 44).

In terms of lifestyle and health-related factors, our findings align with the existing literature showing that high caffeine intake positively correlates with anxiety, consistent with meta-analysis studies confirming that consumption above 400 mg is associated with an elevated risk of anxiety in healthy individuals (18). Likewise, poor sleep is also linked to higher anxiety. Several experimental and longitudinal studies confirm that sleep and anxiety feed on each other as a reciprocal process, hence, poor and inadequate sleep is related to a subsequent increase in anxiety (45, 46). The association between low physical activity and higher anxiety scores is also congruent with the existing literature, which indicates that greater engagement in physical activity, as well as higher exercise intensity, serves as a protective factor against anxiety (47). Interestingly, participants with moderate alcohol consumption experienced a weak association with anxiety in our study, demonstrating the context-dependent nature of this correlation. These findings are consistent with longitudinal studies highlighting that moderate drinking is associated with a lower incidence of anxiety disorders compared to abstinence, whereas heavy drinkers are more prone to depression rather than anxiety (48).

This study promptly contributed to the application of machine learning for predicting anxiety levels. The superior performance of ensemble models, particularly Gradient Boosting and Random Forest, compared to linear models such as Lasso and ElasticNet, was consistent with recent research on the applications of machine learning in psychiatry, where tree-based ensembles frequently outperformed linear regression models due to their ability to capture complex non-linear relationships and higher-order feature interactions. For example, a recent study conducted in China on multi-model machine learning efficacy for predicting depressive symptoms found Random Forest to be the most reliable model, outperforming XGBoost, LightGBM, and Support Vector Machine in terms of precision, accuracy, recall, and F1-score (49). Our findings corroborate recent research on applying machine learning models to predict anxiety and depression symptoms in large population datasets and similarly observed Gradient Boosting as achieving the best performance metrics (50). The comparative underperformance of linear models in our study suggests that anxiety is influenced by multiple complex lifestyle, psychological, and physiological factors, which cannot be adequately captured by linear relationships alone. Another study similarly observed the enhanced performance of Gradient Boosting and Random Forest in predicting stress levels among students, highlighting that ensemble models outperform other state-of-the-art algorithms (51) Collectively, these studies support our findings, suggesting that psychological outcomes such as anxiety are shaped by interdependent and complex predictors that are more precisely captured by ensemble models rather than linear algorithms.

Our study utilized a large dataset, incorporating statistical approaches alongside machine learning validation. The inclusion of multiple domains such as lifestyle, demographic, physiological, and psychological factors allowed for a more comprehensive evaluation of anxiety predictors. Furthermore, integrating traditional statistical methods with machine learning proved helpful in providing converging evidence, ensuring both predictive rigor and interpretability. This study has some limitations. Anxiety levels were assessed using self−reported measures rather than standardized diagnostic interviews, which may underestimate clinical severity. Although the dataset was diverse and large, it was derived from self−reported, observational survey data and is not nationally representative of any specific population, which may limit the generalizability of our findings to clinically diagnosed populations. In addition, all predictors were derived from self−report items, and several key constructs were represented by binary or scalar indicators without detailed clinical information. Likewise, lifestyle and physiological measures were captured using summary indices, which may introduce measurement error and reduce clinical specificity. Finally, the reliance on participants’ retrospective and subjective evaluations is a key limitation, because such reports are susceptible to recall errors and social desirability bias and lack the temporal resolution offered by passive physiological or digital monitoring. Moreover, despite the strong predictive performance of ensemble machine learning models, their interpretability remains limited, highlighting the need for explainable approaches to ensure clinical applicability. Future studies should employ longitudinal designs, validate models in clinically recruited samples, and explore the integration of biological and digital phenotyping measures to strengthen predictive accuracy and support precision mental health care.

## 5 Conclusion

This study highlights the strong influence of lifestyle and psychosocial factors on anxiety particularly stress, sleep duration, and caffeine intake and also demonstrates the potential of ensemble models in predicting anxiety levels by outperforming traditional and single-model approaches. While the findings emphasize the importance of integrating psychological and behavioral factors in mental health research, future validation in clinical settings is needed before translation into practice. These insights may support health professionals in planning early-stage screening strategies to reduce the burden of anxiety. Moreover, integrating machine learning models in future studies could help build clinical trust and promote their adoption in real-world mental health care.

## Data Availability

Publicly available datasets were analyzed in this study. This data can be found here: https://www.kaggle.com/datasets/natezhang123/social-anxiety-dataset/data.
